# 2D Transformations of Energy Signals for Energy Disaggregation

**DOI:** 10.3390/s22197200

**Published:** 2022-09-22

**Authors:** Pascal A. Schirmer, Iosif Mporas

**Affiliations:** 1 School of Physics, Engineering and Computer Science, University of Hertfordshire, Hatfield AL10 9AB, UK; 2 Department of Power Electronics, BMW AG, 80788 Munich, Germany

**Keywords:** appliance identification, Non-Intrusive Load Monitoring (NILM), time series imaging, two-dimensional signal representations

## Abstract

The aim of Non-Intrusive Load Monitoring is to estimate the energy consumption of individual electrical appliances by disaggregating the overall power consumption that has been sampled from a smart meter at a house or commercial/industrial building. Last decade’s developments in deep learning and the utilization of Convolutional Neural Networks have improved disaggregation accuracy significantly, especially when utilizing two-dimensional signal representations. However, converting time series’ to two-dimensional representations is still an open challenge, and it is not clear how it influences the performance of the energy disaggregation. Therefore, in this article, six different two-dimensional representation techniques are compared in terms of performance, runtime, influence on sampling frequency, and robustness towards Gaussian white noise. The evaluation results show an advantage of two-dimensional imaging techniques over univariate and multivariate features. In detail, the evaluation results show that: first, the active and reactive power-based signatures double Fourier based signatures, as well as outperforming most of the other approaches for low levels of noise. Second, while current and voltage signatures are outperformed at low levels of noise, they perform best under high noise conditions and show the smallest decrease in performance with increasing noise levels. Third, the effect of the sampling frequency on the energy disaggregation performance for time series imaging is most prominent up to 1.2 kHz, while, above 1.2 kHz, no significant improvements in terms of performance could be observed.

## 1. Introduction

The aim of Non-Intrusive Load Monitoring (NILM) is to estimate the power consumption on the device level from the aggregated power-consumption signal of a household or a building [1], while minimizing the number of installed energy meters and thus reducing the wiring harness and improving the retrofitting capabilities [1,2]. NILM is defined as a single-channel source-separation task, and the methods that have been proposed in the literature to solve it can be classified into three main categories [3], namely (i) the pattern-matching (elastic matching) approaches which detect load signatures in the aggregated power-consumption signal by comparing them to a set of reference signatures [4,5,6]; (ii) the source-separation methods, including matrix and tensor factorization as well as sparse coding, which separate base components and activations using numeric solvers [7,8,9]; and (iii) the model-based approaches which are based on machine learning algorithms, usually training one model per device, in order to estimate the power consumption of the loads of interest from the aggregated signal [10,11,12].

In detail, within the last few decades, NILM has been employed in many utility and non-utility applications. As regards the utility applications, energy-consumption reduction for residential [13,14] and industrial [15] areas is the most common application. Furthermore, NILM has been used in energy management of smart-grids to optimize load schedules as well as to increase customers’ satisfaction [16,17]. Moreover, NILM has been used to improve load forecasting utilizing specific appliance-usage patterns [11]. In non-utility applications, NILM has been used for fault detection and diagnostics in both the industrial [18] and the residential sectors [19]. Moreover, the privacy-persevering nature of NILM has been used for human behavior monitoring [20]. Moreover, NILM was evaluated in terms of its ability to extract socio-economic information and consumer behavior [21].

The recent development of deep machine learning algorithms and the creation of big data collections have resulted in advanced NILM methodologies. NILM methodologies that are based on Deep Neural Networks (DNNs) [22], Convolutional Neural Networks (CNNs) [15], Long-Short-Term-Memory (LSTM), and Recurrent Neural Networks (RNNs) are presented in the bibliography [23]. Specifically, in [24], the authors presented a causal CNN with gate-dilation optimisation, while, in [25], the authors proposed a concatenated CNN method for high sampling frequencies. In [26], the authors presented a bidirectional LSTM approach with forward and backward path optimization and Bayesian optimization of the hyper-parameter. In [27], the authors proposed the use of RNNs combined with convolutional layers, and, in [28], the authors presented a NILM method based on deep RNNs. Moreover, recently published work on NILM is focused on the use of Generative Adversarial Networks (GANs) [29,30,31] and on bidirectional Transformers [32] in order to use self-attention mechanisms to increase the accuracy of NILM [33], as well as the robustness [30,34] of NILM methods. The transfer capability of NILM methods was studied in [35,36]. Moreover, the areas of fault detection [19] and privacy and security-sensitive Information extraction [3] have been studied.

The above-described approaches utilize either one-dimensional time series as input features [26,37] or multivariate time series of several different features, e.g., active power, reactive power, apparent power, and current, as in [24,27]. However, CNNs in particular were originally proposed as feature-extraction engines for two-dimensional and three-dimensional inputs, e.g., for image processing [38]. Therefore, few approaches have investigated the transformation of one-dimensional time series into two-dimensional signal representations while considering the physical nature of the NILM problem, i.e., considering the harmonic content or the relationship between active and reactive power. For example, in [39], a double-Fourier-integral-based approach for high-frequency energy disaggregation was proposed, and, in [40], a low-frequency approach based on active and reactive power signatures was proposed. Furthermore, voltage and current signatures were used to convert raw measurements into two-dimensional signatures [41,42]. Moreover, in [43,44] time series imaging approaches for univariate time series, i.e., when only a single feature is available, were investigated. However, it is not clear which two-dimensional representations have the best disaggregation performance, since, to the best of the authors knowledge, time-series-imaging techniques have not been compared with each other before. Therefore, in this paper, we investigate the NILM performance of two-dimensional signatures utilizing high- and low-frequency data. Furthermore, we compare the two-dimensional representations with previously published approaches using univariate or multivariate features. The contributions are as follows.

1. Six time-series-imaging (two-dimensional representations) techniques were compared on high- and low-frequency data.
2. The convergence behavior and the influence on the sampling frequency of the two-dimensional representations were evaluated.
3. The robustness to noise for the six evaluated time series imaging approaches was evaluated.

The remainder of the paper is structured as follows. In Section 2, an introduction to time series imaging for energy consumption signals is provided. In Section 3, the evaluated architecture utilizing two-dimensional representations is presented. In Section 4, the experimental setup is provided, and the evaluation results are presented in Section 5. The discussion is provided in Section 6, while the paper is concluded in Section 7.

## 2. Time Series Imaging for Energy-Consumption Signals

Let $x_{agg}\in R^{T}$ and $y_{agg}\in R^{T}$ be two aggregated discrete time signals of length *T* samples, e.g., current ($i_{agg}$) and voltage ($v_{agg}$) for high-frequency measurements or active power ($p_{agg}$) and reactive power ($q_{agg}$) for low-frequency measurements, continuously measured by a smart meter. The two signals are time-aligned (time-synchronous acquisition and in parallel A/D conversion); thus, when each signal is segmented to frames of length *W* samples for any arbitrary frame $x_{agg}^{\tau }\in R^{W}$ of $x_{agg}$, with $x_{agg}^{\tau }=\left[x\left(t_{0}\right),x\left(t_{0}+1\right),\ldots ,x\left(t_{0}+W-1\right)\right]$, there is also a frame $y_{agg}^{\tau }\in R^{W}$ of $y_{agg}$, with $y_{agg}^{\tau }=\left[y\left(t_{0}\right),y\left(t_{0}+1\right),\ldots ,y\left(t_{0}+W-1\right)\right]$, where $t_{0}$ is an arbitrary starting sample of the frame. Based on the above, the following six time series imaging methods are introduced: Voltage–Current (VI) trajectories (in Section 2.1), Double Fourier Integral Analysis (DFIA) (in Section 2.2), active–reactive (PQ) transformation (in Section 2.3), Recurrence (REC) plot (in Section 2.4), Gramian Angular Field (GAF) (in Section 2.5), and Markov Transition Field (MKF) (in Section 2.6).

### 2.1. VI-Trajectory

VI-Trajectories describe the changes of current and voltage in a two-dimensional plane, thus transforming the two time series of the current ($i_{agg}$) and the voltage ($v_{agg}$) into a two-dimensional array. Considering a frame of current $i_{agg}^{\tau }$ and voltage $v_{agg}^{\tau }$, the VI-Trajectory can be calculated as follows [41,42]:

1. Normalize the waveforms to their absolute maximum values, i.e., ${\tilde{i}}_{agg}^{\tau }=\frac{i_{agg}^{\tau }}{\max\left(\left|i_{agg}\right|\right)}$ and ${\tilde{v}}_{agg}^{\tau }=\frac{v_{agg}^{\tau }}{\max\left(\left|v_{agg}\right|\right)}$.
2. Define a uniform grid with grid size $\Delta i=\frac{max(|i_{agg}|)}{W/2}$ and $\Delta v=\frac{max(|v_{agg}|)}{W/2}$.
3. Map the current and voltages samples ${\tilde{i}}_{agg}\left(i\right)$ and ${\tilde{v}}_{agg}\left(j\right)$ with 1≤i,j≤W to the W×W grid cells, obtaining the VI-Trajectory feature vector $F_{i,j}^{\tau }\in R^{W\times W}$, where each element in $F_{i,j}^{\tau }$ indicates if a combination of current and voltage exists or not.

### 2.2. Double Fourier Integral Analysis

In DFIA, an output function f(·) is defined [45] by the cyclically varying signals $x_{agg}^{\tau }$ and $y_{agg}^{\tau }$, i.e., $f(x_{agg}^{\tau },y_{agg}^{\tau })$, i.e., $z_{i,j}^{\tau }=x_{agg}^{\tau }\left(i\right)\cdot y_{agg}^{\tau }\left(j\right)$, with 1≤i,j≤W and $Z^{\tau }\in R^{W\times W}$ being the two-dimensional representation for the τ-th frame. Each two-dimensional plane, $Z^{\tau }$, contains the information of the trajectories in the x/y directions. For energy disaggregation, the double Fourier transform is calculated for each $Z^{\tau }$ frame, i.e.,

(1) $$F_{k,l}^{\tau }=\frac{1}{W^{2}}\sum_{i=1}^{W}\sum_{j=0}^{W}z_{i,j}\cdot e^{-j2\pi (\frac{k}{W}i+\frac{l}{W}j)},$$

where 1≤k<K and 1≤l<L are index variables. The magnitude and/or phase of each feature vector $F^{\tau }\in C^{W\times W}$ is then used as the input of a machine learning model for classification or regression.

### 2.3. PQ Transformation

When high-frequency measurements are not available, the time frames of $x_{agg}^{\tau }$ and $y_{agg}^{\tau }$ can be used to form a two-dimensional PQ-representation. Specifically, if low-frequency active and reactive power are available, power can be calculated as in Equation (2).

(2) $$F_{i,j}^{\tau }=\sqrt{p_{\;agg}^{\tau }{\left(i\right)}^{2}+q_{agg\;}^{\tau }{\left(j\right)}^{2}},$$

where $F_{i,j}^{\tau }\in R^{W\times W}$ with 1≤i,j≤W is the two-dimensional representation of frame τ on the PQ plane, with the diagonal elements of $F^{\tau }$ representing the instantaneous apparent power. Similarly, when the current and voltage are available with high-frequency resolution, a power plane can be calculated as in Equation (3).

(3) $$F_{i,j}^{\tau }=i_{agg}^{\tau }\left(i\right)\cdot v_{agg}^{\tau }\left(j\right)$$

### 2.4. Recurrence Plot

The above-described methods have utilized both time series, $x_{agg}$ and $y_{agg}$, to compute the two-dimensional representation that can be used as input features. However, some datasets offer only univariate measurements, e.g., only active power or current [46], thus not allowing the utilization of VI, DFIA, or PQ approaches. A method to utilize univariate time series imaging is the recurrence plot, which computes the pairwise distances between two successive frames. Based on the definition of the frame $x_{agg}^{\tau }$, the recurrence plot can be calculated as in Equation (4).

(4) $$F_{i,j}^{\tau }=\Theta \left(\epsilon -\left|\right|x_{agg}^{\tau }\left(i\right)-x_{agg}^{\tau }\left(j\right)\left|\right|\right),$$

where $F_{i,j}^{\tau }\in R^{W\times W}$ with 1≤i,j≤W is the two-dimensional recurrence plot representation, Θ is the Heaviside function, and ε is a threshold parameter.

### 2.5. Gramian Angular Field

Similarly, Gramian Angular Fields (GAFs) can create two-dimensional representations based on a single time series. In detail, the GAF is a method creates a matrix of temporal correlations for each $\left(x_{i},x_{j}\right)$ within a frame $x_{agg}^{\tau }\in R^{W}$. First, the time series is rescaled in the range [$x_{min}$, $x_{max}$] with $-1\leq x_{min}<x_{max}\leq 1$ as described in Equation (5).

(5) $${\tilde{x}}_{i}^{\tau }=x_{min}+\left(x_{max}{-x}_{min}\right)\cdot \frac{x_{i}^{\tau }-min\left(x^{\tau }\right)}{max\left(x^{\tau }\right)-min\left(x^{\tau }\right)},$$

where ${\tilde{x}}_{i}^{\tau },\forall i\in 1,\ldots ,W$, is the rescaled *i*-th sample of the τ-th frame of $x_{agg}$. After rescaling ${\tilde{x}}_{i}^{\tau }$ can be represented in polar coordinates based on the polar coordinates’ angle, $\phi_{i}^{\tau }=arcos\left({\tilde{x}}_{i}^{\tau }\right)$, the Gramian Angular (Summation) Field can be calculated as in Equation (6).

(6) $$F_{i,j}^{\tau }=\cos\left(\phi_{i}+\phi_{j}\right),$$

where $F_{i,j}^{\tau }\in R^{W\times W}$ with 1≤i,j≤W is the two-dimensional representation of the GAF.

### 2.6. Markov Transition Field

The Markov Transition Field (MTF) discretizes a time series into *Q* bins and spreads out the temporal information after calculating its Markov matrix. Considering the time frame $x_{agg}^{\tau }$, each sample $x_{agg}^{\tau }\left(i\right)$ is assigned to one bin $q_{j}$ with jϵ[1,Q] creating an Q×Q adjacent matrix which counts the transitions among the quantile bins, where $w_{i,j}$ denotes the frequency of a point of quantile $q_{j}$, following a point of quantile $q_{i}$. Based on this, the MTF can be written as in Equation (7).

(7) $$F_{i,j}^{\tau }=\left[\begin{array}{ccc} w_{\left.i,j\right|x_{1}\in q_{i},x_{1}\in q_{j}} & \cdots & w_{\left.i,j\right|x_{1}\in q_{i},x_{W}\in q_{j}}\\ \vdots & \ddots & \vdots \\ w_{\left.i,j\right|x_{W}\in q_{i},x_{1}\in q_{j}} & \cdots & w_{\left.i,j\right|x_{W}\in q_{i},x_{W}\in q_{j}} \end{array}\right],$$

where $F_{i,j}^{\tau }\in R^{W\times W}$ with 1≤i,j≤W is the two-dimensional representation of the MTF.

**Figure 1:**
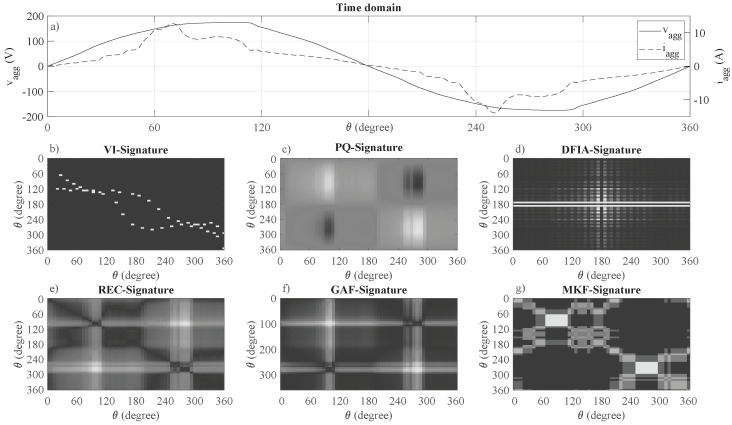
One electrical cycle for the aggregated current and voltage (**a**) as well as the transformed signals as obtained from the time series imaging (**b**–**g**).

A graphical illustration example of one electrical cycle of the current and voltage of the aggregated signal, as well as the graphical representation of the six methods for time series imaging, is illustrated in Figure 1.

As can be seen in the example of Figure 1, the methods for imaging time series into two-dimensional signatures result in very different two-dimensional representations of the one-dimensional voltage and current signals. In detail, the VI-Trajectory and DFIA result in relatively sparse two-dimensional patterns, whereas all other approaches have densely filled matrices. Moreover, due to the periodically varying current and voltage signals, the two-dimensional representations show symmetry properties for approaches (b–f), while the MTF representations are more chaotic due to the discretization of the data and putting the data into different bins.

## 3. NILM Using Two-Dimensional Signal Representations

NILM is defined as the problem of extracting the power consumption on the device level from the aggregated signal measured by one sensor in short-time analysis, i.e., in time-sliding windows. Specifically, given a set of *M*-1 known appliances with each of them consuming power $p_{m}$ with 1≤m≤M−1, the aggregated power $p_{agg}$ measured by the sensor is:

(8) $$p_{agg}=f\left(p_{1},p_{2},\ldots ,p_{M-1},e\;\right)=\sum_{m=1}^{M-1}p_{m}+e=\sum_{m=1}^{M}p_{m},$$

where $e=p_{M}$ is the noise generated by one or more unknown device, and f(·) is the aggregation function. In NILM, the goal is to find estimations ${\hat{p}}_{m}$, $\hat{e}={\hat{p}}_{M}$ of the power consumption of each device *m* using a disaggregation function $f^{-1}\left(\cdot \right)$ with minimal estimation error, i.e.,

(9) $${\hat{P}=\left\{{\hat{p}}_{1},\;{\hat{p}}_{2},\ldots ,{\hat{p}}_{M-1},\hat{e}\right\}=f^{-1}(p}_{agg}).$$

Given that Equation (9) cannot practically be solved analytically, most NILM approaches perform short-time analysis by segmenting the aggregated signal into frames and then estimating the power consumption of each appliance for every frame. In order to feed the disaggregation function $f^{-1}\left(\cdot \right)$ with more distinctive information, every frame of the active power signal, $p_{agg}^{\tau }$ (or $i_{agg}^{\tau }/v_{agg}^{\tau }$ for high-frequency data), is usually transferred to a feature representation, $F_{agg}^{\tau }$, as discussed in Section 2. Based on this, the disaggregation problem from Equation (9) can be reformulated on the frame level using the feature vectors as defined in Section 2.

(10) $${{\hat{P}}^{\tau }=\left\{{\hat{p}}_{1}^{\tau },\;{\hat{p}}_{2}^{\tau },\ldots ,{\hat{p}}_{M-1}^{\tau },{\hat{e}}^{\tau }\right\}=f^{-1}(F}_{agg}^{\tau })$$

**Figure 2:**
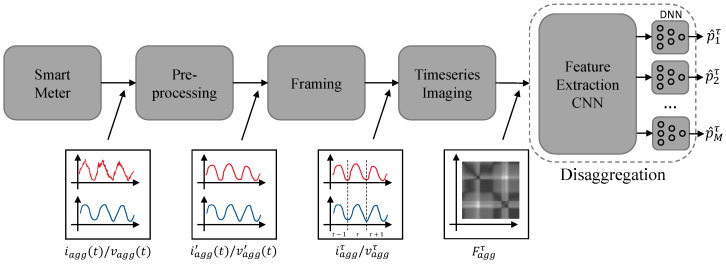
Non-Intrusive Load-Monitoring architecture using time series imaging for high-frequency data inputs.

The architecture of the presented NILM method using time series imaging consist of four steps, namely pre-processing, framing, time series imaging (two-dimensional signal representation) as discussed in Section 2, and disaggregation. The architecture for high-frequency data inputs is illustrated in Figure 2.

As can be seen in Figure 2, the raw current ($i_{agg}\left(t\right)$) and raw voltage ($v_{agg}\left(t\right)$) signals are initially pre-processed. Pre-processing consists of two steps, namely filtering and down-sampling of the data, resulting in the pre-processed signals $i_{agg}^{\prime }\left(t\right)$ and $v_{agg}^{\prime }\left(t\right)$. After pre-processing the signals are frame blocked into frames of length *W* resulting into the signals $i_{agg}^{\tau }\in R^{W}$ and $v_{agg}^{\tau }\in R^{W}$. Finally, time series imaging is performed generating the two-dimensional frame representation $F_{agg}^{\tau }$, which is used as input to the disaggregation stage. The disaggregation stage, consisting of a CNN operating as a feature-extraction engine and a DNN estimating the appliance consumption ${\hat{p}}_{m}^{\tau }$.

## 4. Experimental Setup

The NILM architecture based on the imaging time series as described in Section 3 was evaluated using the datasets and regression algorithm presented below.

### 4.1. Datasets

The proposed architecture was evaluated using two different datasets, namely the REDD [46] and the AMPds2 [47], and was chosen for two reasons. First, REDD is the most widely used dataset in the energy disaggregation task and provides, next to low-frequency measurements (1 Hz), high-frequency measurements (16.5 kHz) for the aggregated signal, including both current and voltage signals [46]. Furthermore, the high-frequency measurements enable the comparison of different sampling rates and their impact on the disaggregation performance. Second, AMPds2 was chosen as it enables one-to-one comparison to previously proposed architectures such as WaveNILM [24] or SSHMM [10] since there are no missing data points and all data are time-synchronized [47]. Brief descriptions of the datasets can be found in Table 1, where NAR indicates the noise-to-aggregate ratio as defined in [48].

As regards the AMPds2 dataset, in the literature, either all or a subset of appliances are used, often referred to the deferrable loads; thus, in the evaluation protocol and the experimental results, we have included both scenarios. In specific, the deferrable loads for the AMPds2 dataset are the cloth dryer, the dish washer, the HVAC system, the heat pump, and the kitchen wall oven [10].

### 4.2. Pre-Processing

The data were pre-processed as commonly conducted previously in the literature [47] using median filtering with a filter length of 21 samples. Furthermore, the high-frequency data were down-sampled by a factor of five, resulting in an aggregated signal with sampling frequency of 3.3 kHz, enabling the usage of the first 27 harmonics of the current and the voltage, which are capturing most of the information as discussed in [49,50]. Similarly, the data at appliance level were down-sampled to a resolution of one minute to be aligned with the previously published literature [10,51,52]. Both aggregated and appliance data were normalized using mean-std normalization, while the mean value and the value of the standard deviation (std) were calculated using the training data subset only [53]. The normalization procedure is described in Equation (11).

(11) $$\tilde{x}=\frac{x-{\bar{x}}_{train}}{\sigma_{train}},$$

where *x* is the original signal, ${\bar{x}}_{train}$ is the mean value of the signal in the training set, $\sigma_{train}$ is the standard deviation of the signal in the training set, and $\tilde{x}$ is the normalized data.

### 4.3. Model Parametrization

**Figure 3:**
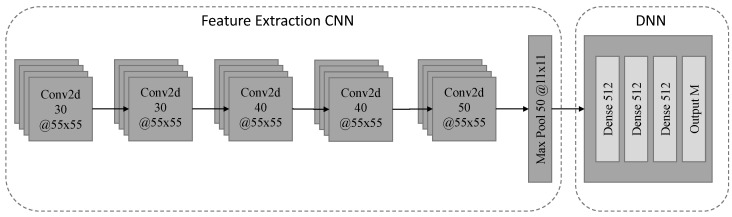
Architecture of the evaluated two-stage NILM model utilizing a two-dimensional CNN for feature extraction and a three layer DNN for regression. For each layer, the number of filter/pooling operations (*X*) and the two-dimensional filter/pooling size (Y×Z) is given as (X@Y×Z).

A two-dimensional CNN was utilized at the regression step as in [53] with RELU activation functions in the intermediate layers and with a linear activation function in the last one. In addition, the one-dimensional kernels were replaced by two-dimensional kernels, to account for the two-dimensional inputs through the imaging of the time series’ of the proposed method. Furthermore, the number of layers and neurons in the dense part of the model have been selected after hyper-parameter optimization. The detailed layer setup, including the number of filters is illustrated in Figure 3.

The NILM models were trained using TensorFlow, and the Keras backend was utilised with Adam optimisation during the training of the models (The Python implementation of the architecture is available at https://github.com/pascme05/HF_NILM, accessed on 20 August 2022). The selected values of the hyper-parameters of the models and the parametrization of the Adam optimisation are shown in Table 2.

As can be seen in Table 2, different frame lengths have been utilized for the high-frequency (HF) and the low-frequency (LF) experiments. In detail, for HF experiments, at least one fundamental period (55 samples @3.3 kHz) of the electrical signals must be captured to calculate the harmonic spectrum or the VI trajectories. Conversely, for LF signals, there is no such restriction; thus, a frame length of 30 min (i.e., 30 samples) has been used since it has shown good results in previous work [40]. Similarly, 100 epochs were used for training the HF CNN due to the higher number of data samples, while 50 epochs were used for the LF CNN.

### 4.4. Experimental Protocols

The proposed architecture was evaluated using three different experimental protocols. Specifically, the evaluation was performed using 10-fold cross-validation with 10% of the training data being used for validation, thus the test data are completely unseen in training unlike a previous approach [24], where the validation data were used for testing as well. Furthermore, all scenarios are utilizing the so-called noisy configuration [10] without modelling the unrecorded data as an additional ghost appliance. A tabulated overview of all three evaluated protocols is provided in Table 3.

As can be seen in Table 3, protocol #1 evaluates the performance on HF data by utilizing the REDD dataset. Protocol #2 and protocol #3 are used for direct comparison with the literature evaluating the performance in the two most common scenarios when utilizing AMPds2, namely through the evaluation of all (ALL) loads and the deferral (DEF) loads, using current as the output feature [10].

## 5. Experimental Results

The NILM methodology described in Section 3 was tested using the experimental setup presented in Section 4. For the purpose of accurate comparison, performance was tested in terms of estimation accuracy ($E_{ACC}$), as proposed in [46].

(12) $$E_{ACC}=1-\frac{\sum_{\tau =1}^{T}\sum_{m=1}^{M}\left|{\hat{p}}_{m}^{\tau }-p_{m}^{\tau }\right|}{2\sum_{\tau =1}^{T}\sum_{m=1}^{M}\left|p_{m}^{\tau }\right|},$$

where ${\hat{p}}_{m}$ is the estimated power of device *m*, with 1<=m<=M, *T* is the number of disaggregated frames, and *M* is the number of disaggregated devices. Furthermore, to compare with previously published approaches, additional accuracy metrics, namely the Mean Absolute Error (MAE) and the normalized Signal Aggregated Error (SAE) are used, which are defined as:

(13) $$MAE=\frac{1}{T}\sum_{\tau =1}^{T}\left|{\hat{p}}_{m}^{\tau }-p_{m}^{\tau }\right|$$

(14) $$SAE=\frac{\left|{\hat{E}}^{m}-E^{m}\right|}{E^{m}},$$

where $E^{m}$ denotes the total energy consumption of the appliance *m* and ${\hat{E}}^{m}$ its predicted value. The results for REDD-3 and REDD-5 are tabulated for the six different two-dimensional transformation methods in Table 4. The results are reported in terms of $E_{ACC}$, MAE, and SAE using active power as the output feature.

As can be seen in Table 4, the PQ-transformed signals outperform all other approaches at REDD-3 dataset, while achieving very high performances at the REDD-5 as well. Conversely, MKF is achieving very low performance for all accuracy metrics in both REDD-3 and REDD-5, while REC and GAF are achieving quite similar results for all accuracy metrics and in both datasets. Similarly, the results for AMPds2 are reported for five different two-dimensional transformation methods, since VI trajectories cannot be calculated due to the missing voltage information in the AMPds2 dataset. The results are reported in terms of $E_{ACC}$, MAE, and SAE using current as output feature and can be found in Table 5.

As can be seen in Table 5, once again PQ transformed signals are outperforming all other methods for both deferrable (protocol #3) and all loads (protocol #2), achieving a maximum accuracy of 94.78%. Furthermore, it can also be seen that MKF is reporting the worst performance for all accuracy metrics for both deferrable and all loads. Moreover, it can be seen that the time series imaging methods that are utilizing two input features, e.g., PQ transformed signals or DFIA, are outperforming methods that utilize only univariate input signals, e.g., REC, GAF, and MKF.

To compare the proposed time series imaging methods to previously proposed approaches, the five approaches reporting the best performance on the AMPds2 dataset have been used for comparison and are tabulated in Table 6. It must be noted that, for the purpose of the direct comparison, the results in Table 6 have been calculated following the evaluation setups of the corresponding articles, e.g., using only the first year of AMPds2 when comparing to [10,24] or using a reduced amount of data (training: 18 August 2012–13 April 2013, testing: 17 May 2013–17 June 2013) when comparing to [26,29,30].

As can be seen in Table 6, the PQ-transformed signals are outperforming most of the other techniques showing the advantages of utilizing two-dimensional representations in combination with CNNs. Only the WaveNILM [24] approach using all appliances is performing 0.2% better than the PQ-transformed signals; however, that approach is utilizing four input features (active power, reactive power, apparent power, and current), while the PQ transformation approach is only utilizing two input features (active power and reactive power). Furthermore, when comparing only three appliances as in [26,29,30], the PQ transformation is achieving significantly better performances.

## 6. Discussion

Further to the experimental results presented in Section 5, three topics, namely the influence on the convergence and runtime of the two-dimensional representation methods (Section 6.1), the influence on the sampling frequency (Section 6.2), and the robustness towards noise (Section 6.3), are discussed in this Section.

### 6.1. Runtime and Convergence

**Figure 4:**
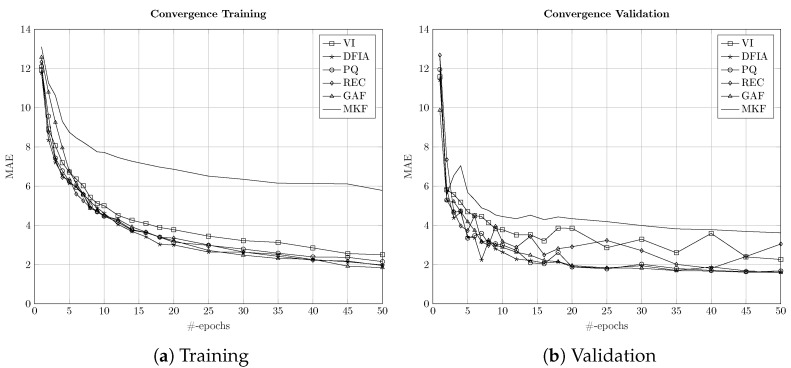
Convergence of the six time series 2D representation methods for 50 epochs of training using the REDD database during (**a**) training and (**b**) validation.

For sequence-to-point approaches with high dimensionality of the input data, as in the proposed one using time series imaging with two-dimensional representations, algorithm convergence and real-time capability are of significant interest [35,39]. Therefore, the convergence behavior for a fixed size of data and the execution times per sample were investigated. The comparison of the convergence of protocol #1 of the six different two-dimensional representation methods is shown for the training and the validation for the first 50 epochs in Figure 4.

As can be seen in Figure 4, all the two-dimensional representation methods are showing good convergence for both training and validation. In detail, the four approaches, DFIA, PQ, REC, and GAF, are showing rather similar convergence behavior, which is reflected in their similar performances tabulated in Table 4. Conversely, VI trajectories are not converging the as-good, which was reflected in their slightly worse performance during disaggregation. Furthermore, MKF is showing significantly worse convergence behavior, which is in line with the results reported in Section 5 for both high- and low-frequency data. Additionally, the convergence behavior for real-time applications and the runtime of the algorithms are crucial. The execution time per sample for the six different time series imaging methods was calculated on an AMD Ryzen 3700 CPU with 32 GB RAM. The results are shown in Table 7.

As can be seen in Table 7, the PQ transformation outperforms all other approaches due to only relying on one matrix multiplication of the current and voltage frame, whereas DFIA requires a frequency two-dimensional Fourier analysis of a W×W size matrix. Similarly, all other approaches require significantly more multiplications and additions to calculate the corresponding signatures, as described in Section 2, thus reporting higher execution times.

### 6.2. Sampling Frequency

**Figure 5:**
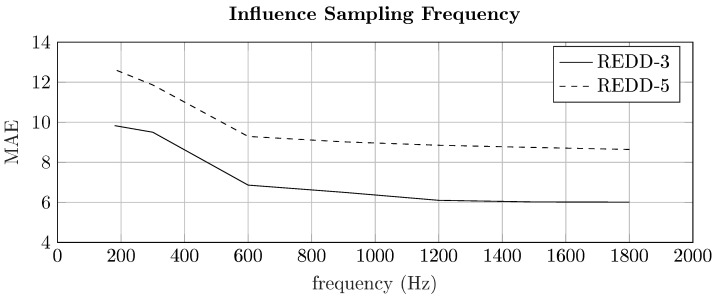
Performance for the REDD database for different sampling frequencies using PQ-transformed signals.

To design cost-effective smart meter hardware, it is important to understand the dependencies of the disaggregation performance on the sampling frequency. Furthermore, significant influences of the sampling frequency on the disaggregation performance have been previously reported in the literature [54,55]. To investigate the dependency of the performance on the sampling frequency, the high-frequency part of the REDD database was used. In detail, the impact of the sampling frequency on the NILM performance was investigated between 180 Hz and 1800 Hz, capturing up-to the 15th harmonic when considering a line frequency of 60 Hz. The performance for different sampling frequencies in terms of MAE is illustrated in Figure 5.

As can be seen in Figure 5, the MAE decreases while the sampling frequency increases, due to the increase of the information captured by the higher order harmonics. However, it can also be seen that an increase above 1200 Hz does not further decrease the MAE significantly. Since the amplitude of the higher-order harmonics decreases with the square of the harmonic order, i.e., a fundamental current amplitude of the maximum line current per phase (15 A) corresponds to the amplitude of the 10th harmonic, which is approximately equal to $\frac{15\;A}{100}=150$ mA, the information captured by the high-order harmonics is decreasing quickly not providing any additional information to the disaggregation algorithm [41]. Moreover, due to the electrical design of certain appliances, especially the odd order harmonics are having a significant influence on the performance, which can be seen in the decrease around 360 Hz (3rd harmonic), 600 Hz (5th harmonic), and 840 Hz (7th harmonic). It should also be noted that REDD-3 and REDD-5 show very similar characteristics in terms of the influence of the sampling frequency to the NILM performance, which is due to the underlying fundamental physical properties of the current harmonics [49,56].

### 6.3. Robustness to Noise

**Figure 6:**
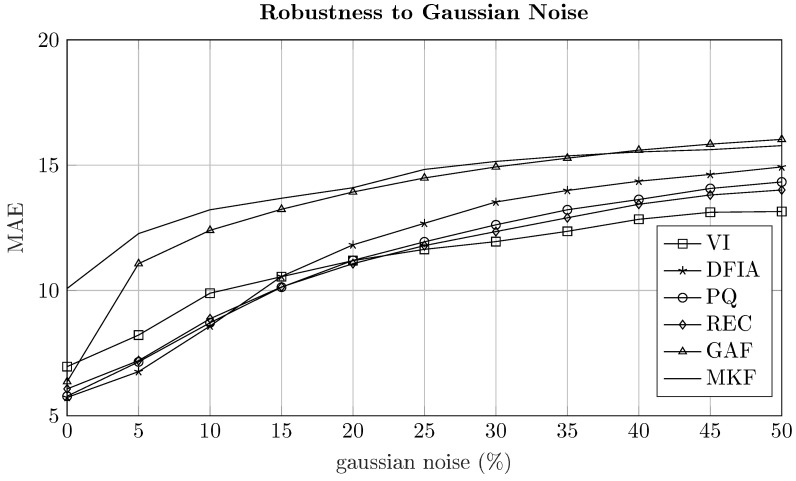
Influence of the noise level on the performance of energy disaggregation for different time series imaging methods.

To investigate how robust the two-dimensional representation methods are against noise, additive white noise was added to the aggregated signal during testing. In detail, different levels of white noise have been investigated, namely 0–50%, with the noise level broken into increments of 5%. Similarly to [30], a noise level of x% means that the aggregated signal during testing has a deviation of x% when being compared to the original signal. The influence on the MAE when adding a certain level of noise is illustrated for the six different two-dimensional representation methods in Figure 6. To account for influences during testing and training, each of these results was calculated using 5-fold cross-validation and 50 epochs for training.

As can be seen in Figure 6, there are differences considering the NILM performance under different levels of noise. First, even though the five two-dimensional representation methods (VI, DFIA, PQ, REC, and GAF) have shown very similar performances under noiseless conditions, their behavior under noisy conditions is different. While, for low noise conditions (0–10%), DFIA shows the best performance, which has already been reported in [39], VI, PQ, and REC signatures outperform DFIA for noise levels larger than 15%. This is probably since DFIA is a technique working in the frequency domain, thus relying on the correct identification of device-specific harmonics for disaggregation. Furthermore, while VI signatures have the worst performance in noiseless conditions, the increase of MAE with increasing noise is the smallest, reporting the best results for high noise conditions (>25%). This robustness to noise is probably due to a sparse representation of the current and voltage waveform that the VI trajectories are having, which cannot easily be distorted by adding white noise (see Figure 1b). Moreover, GAF does not perform well under noisy conditions, reporting an increase of MAE by approximately 75% when adding 5% of white noise. This is probably due to the fact that GAF is based on an evaluation of phase angles, which are far more sensitive to noise than amplitudes. Second, even though MKF reports the worst absolute performance, the increase in MAE is the smallest for all two-dimensional representation methods, which is probably observed because the MKF is evaluating the signal changes and their frequency of change rather than the signal itself. Therefore, only the changes of devices states are captured, which cannot easily be changed by adding white noise.

## 7. Conclusions

A comparison of time series two-dimensional representation methods for energy disaggregation has been presented. In detail, six different time series 2D representation methods for high-frequency data were investigated, and it was shown that PQ transformations outperform other two-dimensional representation-based approaches in almost all investigated evaluation protocols in terms of NILM performance, as well as in terms of run-time. However, when high-noise conditions are considered current and voltage-based signatures (i.e., VI-trajectories) outperform all other approaches showing the highest robustness against increasing levels of noise. Furthermore, it was shown that approaches utilizing both current and voltage time series (such as VI-trajectories, PQ-transforms, or DFIA) are outperforming approaches that rely on univariate data, i.e., the two-dimensional representation that utilize only one time series (such as REC, GAF, or MTF). Moreover, it was shown that utilizing high-frequency data significantly decreases the energy disaggregation error. However, it was also shown that sampling frequencies above 1200 Hz barely show any improvement on the energy disaggregation performance. In general, the results indicate that two-dimensional representations in combination with CNNs are a suitable choice for addressing the energy disaggregation problem. When comparing with previously published methods, it was shown that two-dimensional signatures achieve equal or better performances, while often using fewer features. In the future, the following research directions should be considered. First, an in-depth investigation on sampling frequencies and their influence on appliance specific disaggregation accuracies should be conducted to obtain a better understanding of the impact of certain harmonics on the disaggregation problem. Second, two-dimensional representations (time series imaging) should be combined with transfer learning, utilizing the transfer capability of pre-trained big CNN models mostly used in computer vision tasks.

## Figures and Tables

**Table 1:** Short description of the REDD and AMPds2 database.

Name	House	Country	Appliances	Sampling Rate	NAR
**REDD**	3, 5	US	19, 22	16.5 kHz	11.1–31.8%
**AMPds2**	-	CA	22	60 sec	17.8

**Table 2:** Hyper-parameter values of the CNN model and parameters of the Adam optimizer. HF and LF are the parameters for using high-frequency and low-frequency data, respectively.

Parameter	Value HF	Values LF
**Input size**	55 × 55	30 × 30
**Batch size**	50	1000
**Epochs**	100	50
**Patience**	15	10
**Learning rate**	0.001	0.001
**Beta-1**	0.9	0.9
**Beta-2**	0.999	0.999
**Epsilon**	$1\times 10^{8}$	$1\times 10^{8}$

**Table 3:** Three experimental protocols including train/test splits and evaluated appliances.

Protocol	Dataset	Model	Appliances	Train	Validation	Test
**#1**	REDD	HF-CNN	ALL	90%	10% of Train	10%
**#2**	AMPds2	LF-CNN	ALL	90%	10% of Train	10%
**#3**	AMPds2	LF-CNN	DEF	90%	10% of Train	10%

**Table 4:** Results for protocols #1 in terms of $E_{ACC}$, MAE, and SAE for the high-frequency data of REDD-3/5.

2D Method	REDD-3 HF			REDD-5 HF		
	*E_ACC_*	MAE	SAE	*E_ACC_*	MAE	SAE
**VI**	83.66%	6.69	0.053	65.62%	13.32	0.655
**DFIA**	85.31%	5.73	0.077	73.10%	10.40	0.036
**PQ**	86.26%	5.60	0.068	76.33%	9.73	0.055
**REC**	84.21%	6.16	0.077	76.51%	9.35	0.098
**GAF**	84.38%	6.30	0.074	77.43%	9.16	0.099
**MKF**	75.50%	9.74	0.074	67.25%	13.24	0.013

**Table 5:** Results for protocols #2 and #3 in terms of $E_{ACC}$, MAE, and SAE for the low-frequency data of AMPds2.

2D Method	AMPds2 ALL			AMPds2 DEF		
	*E_ACC_*	MAE	SAE	*E_ACC_*	MAE	SAE
**DFIA**	80.85%	0.22	0.246	81.84%	0.31	0.263
**PQ**	89.94%	0.12	0.048	94.78%	0.09	0.048
**REC**	80.91%	0.22	0.216	80.04%	0.34	0.352
**GAF**	79.18%	0.24	0.273	77.86%	0.38	0.408
**MKF**	77.94%	0.25	0.311	77.49%	0.39	0.423

**Table 6:** Comparison of the best-performing proposed 2D transformation method (PQ) with state-of-the-art performances reported in the literature in terms of $E_{ACC}$ and MAE (* the approach utilizes the next-to-active and reactive power and current and apparent power as its input features).

2D Method	PQ	SSHMM [10]	WaveNILM [24]	BiLSTM [26]	EnerGAN [29]	EnerGAN++ [30]
**DEF**	95.2%	94.0%	94.7%	-	-	-
**ALL**	90.0%	-	90.2% *	-	-	-
**DR,HO,WO**	13.2	-	-	38.6	35.3	38.5

**Table 7:** Average Execution Time (AET) per sample for different time series imaging approaches.

Imaging Method	VI	PQ	DFIA	REC	GAF	MKF
**AET**	670 us	33 us	170 us	950 us	920 us	980 us

## Data Availability

Not applicable.
